# Assessing the impact of a shadowing programme on in-hospital mortality following trainee doctors’ changeover

**DOI:** 10.1186/s12913-021-06578-y

**Published:** 2021-06-07

**Authors:** Balinskaite Violeta, Bottle Alex, Aylin Paul

**Affiliations:** School of Public Health, Imperial College London, London, AZ SW7 UK

**Keywords:** In-hospital mortality, Trainee doctors’ changeover, Programme evaluation

## Abstract

**Background:**

To assess the impact on seven-day in-hospital mortality following the introduction in 2012 of a shadowing programme for new UK medical graduates requiring them to observe the doctor they are replacing for at least 4 days before starting work.

**Methods:**

Data on emergency admissions were derived from Hospital Episode Statistics between 2003 and 2019. A generalised estimating equation model was used to examine whether the introduction of the programme was associated with a change in mortality.

**Results:**

There were 644,018 emergency admissions, of which 1.8% (7612) ended in death in hospital within a week following the admission. Throughout the study period, there was an annual increase in the number of emergency admissions during July and August, though in-hospital mortality rates declined. The generalised estimating equation analysis found no significant change in the odds of death within 7 days after admission for patients admitted on the first Wednesday in August compared with patients admitted on the last Wednesday in July (OR = 1.03, 95% CI 0.94–1.13, *p* = 0.53). Furthermore, there was no significant change observed for any clinical diagnosis category following the introduction of the shadowing programme.

**Conclusion:**

There was a rising trend in the number of emergency admissions over the study period, though mortality was decreasing. We found no significant association between the introduction of shadowing programme and in-hospital mortality; however, lack of power means that we cannot rule out a small effect on mortality. There are other outcomes that might have changed but were not examined in this study.

**Supplementary Information:**

The online version contains supplementary material available at 10.1186/s12913-021-06578-y.

## Introduction

In the United Kingdom, newly qualified doctors begin their residency training on the first Wednesday in August and has been grimly referred to as “Black Wednesday”, while in North America it happens in July and is known as the “July Phenomenon” [1, 2]. Interest in the potential adverse effects on patient care during this changeover attracted a significant amount of research in the United States [3]. In England, only three studies investigated the association between changeover and adverse patient outcomes, and mixed results were found [4–6]. In early work, Aylin et al. found no significant change in mortality but were only able to examine numbers of death registrations rather than hospital mortality rates [4]. Shuhaiber and colleagues looked at the effect of cardiothoracic resident turnover on cardiac surgical outcomes and found a 30% higher odds of in-hospital mortality after a complex cardiac operation [5]. A later study using administrative hospital data found evidence that patients admitted on the first Wednesday in August had a higher risk of early death compared with patients admitted on the last Wednesday in July [6]. Furthermore, higher mortality risk was observed for patients admitted with a primary medical (rather than surgical or cancer) diagnosis. As a direct consequence of the latter study, in 2012 the Medical Director of the National Health Service (NHS) introduced a shadowing programme. The aim of the programme was to ensure that those entering the foundation year 1 doctor (FY1) posts gain practical familiarity with the specific clinical environment they will be working in [7–9]. The expectation was that such experience directly improves patient safety and benefits the wider functioning of the team, allowing other staff to be included while ensuring adequate staffing of wards and clinics. Though the shadowing programme was compulsory, there was no national curriculum, although guidance was provided to hospital trusts for the induction period (at least 4 days of the induction) [10]. Due to the latter, the form of the inductions carried out varied between trusts: from 4 days to a 2-week programme.

In this follow-up study, we aimed to investigate whether the introduction of the shadowing programme intervention was associated with a change in the in-hospital mortality for patients admitted on the day of the changeover (first Wednesday in August) compared with patients admitted on the previous Wednesday in July using the same methodology as our previous study [6].

## Methods

Patient-level data was extracted from Hospital Episode Statistics (HES) between 2003 and 2019. It is an administrative database that contains information on all admissions to English NHS hospital trusts (trusts can comprise multiple hospital sites). Each patient record contains demographic information (e.g. age, sex and socioeconomic deprivation), the episode of care (for example, date of admission) and diagnosis. Diagnoses are recorded using the International Classification of Diseases, 10th edition (ICD-10). Each patient episode was linked into ‘spells’ (admissions to one provider) and spells were linked into ‘superspells’ (which combine any interhospital transfers). Two patient cohorts were identified: emergency patients admitted on the last Wednesday in July (used as controls) and emergency patients admitted on the first Wednesday in August. We included only acute trusts (168 trusts) that each year take on trainee doctors on the first Wednesday in August. All admissions were divided into three clinical categories based on the Agency for Healthcare Research and Quality’s 259 Clinical Classification System (CCS) diagnostic groups: medical, surgical and neoplasm.

The outcome of interest was all-cause seven-day in-hospital mortality in patients admitted on the first Wednesday in August (changeover group) compared with patients admitted on the last Wednesday in July (control group).

Descriptive data were summarised as total numbers and percentages, grouped by the day of admission and pre/post-shadowing programme (intervention). The crude and adjusted odds ratios and their 95% confidence intervals before and after the shadowing programme were estimated using multilevel logistic regression [11]. The following patient-level variables were included in the regression model: age (six-year bands: 0–14, 15–44, 45–64, 65–74, 75–84, 85+), gender, small-area socioeconomic deprivation (Carstairs quintile) [12], Charlson score (co-morbidities score, NHS adaptation) [13], year and week of admission.

A generalised estimating equation (GEE) with autoregressive correlation structure model was used to examine how in-hospital mortality changed over time and how it was affected by the introduction of the shadowing programme [14, 15]. The model included binary variables for group (1 – if patient was admitted on the last Wednesday in July (control group), 0 – if patient was admitted on the first Wednesday in August (changeover group)) and intervention (1 – after shadowing programme, 0 – before shadowing programme), fixed effects for time, and random trust-level effects to adjust for the dependency of observations within each trust. In addition, the model was adjusted by patient-level variables such as age, gender, small-area socioeconomic deprivation, Charlson comorbidity score, year and week of admission. A *p*-value less than 0.05 was considered statistically significant, and all analysis was carried out with SAS 9.4 software package (SAS Institute, Cary, N.C., USA).

### Ethical considerations

Ethical approval was obtained from the London-South East Ethics Committee (REC ref. 15/LO/0824) to use data for research and measuring quality of delivery of healthcare.

## Results

**Fig. 1:**
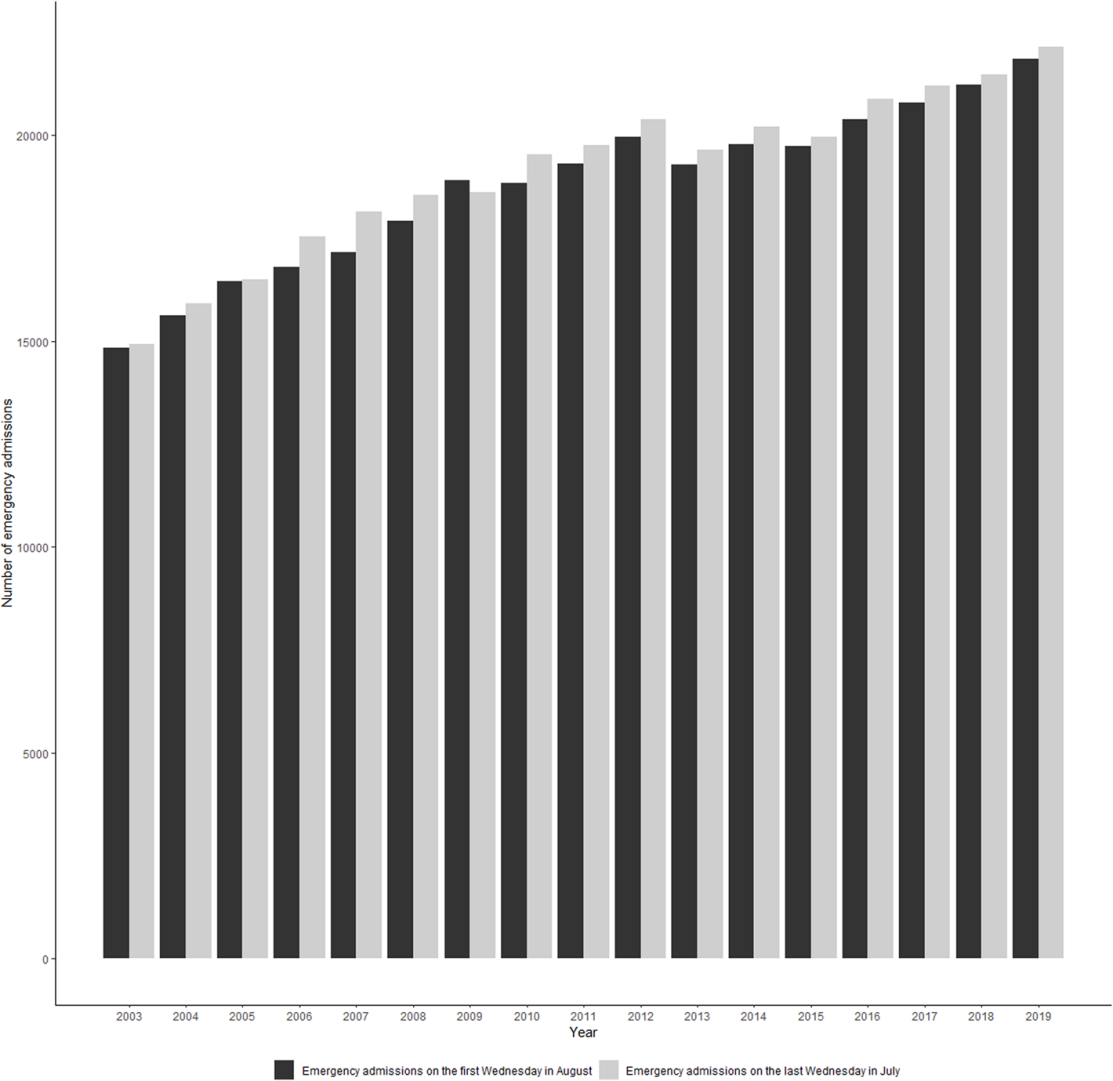
Number of emergency admissions in acute NHS trusts in England

**Fig. 2:**
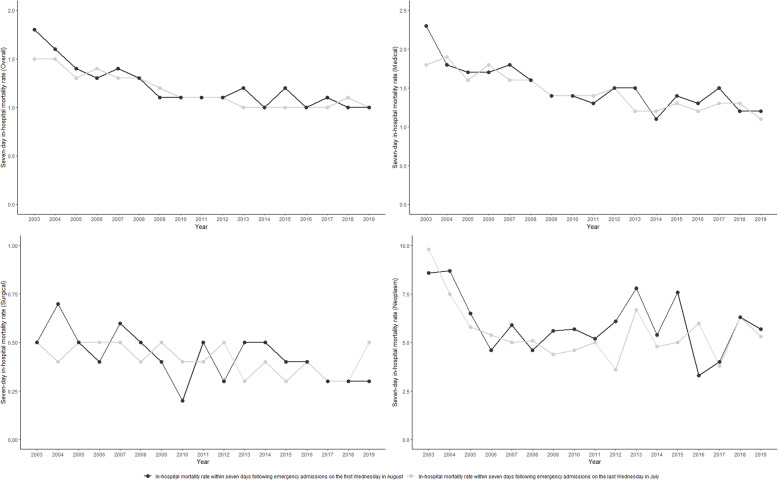
Crude in-hospital mortality rates in England between 2003 and 2019

During the study period, 644,018 emergency admissions were extracted (325,307 occurred on the last Wednesday in July and 318,711 occurred on the first Wednesday in August). Over 1.2% (7612) of these admissions ended in death in hospital within a week following the admission Table S1 (see Additional file 1). Throughout the study period, there was a steady increase in emergency admissions during July and August, though in-hospital mortality rates declined (Figs. 1 and 2). There was a small difference in the characteristics of patients admitted on the last Wednesday in July compared with patients admitted on the first Wednesday in August. However, comparing patient characteristics before (2003 to 2011) and after (2012 to 2019) the shadowing programme was implemented, there was a higher proportion of older and comorbid patients admitted to hospital after the intervention. There was no significant difference in unadjusted seven-day in-hospital mortality rates (1.2% vs 1.2%, *p* = 0.47). No significant differences in unadjusted mortality rates were observed for any clinical diagnosis category (medical: 1.5% vs 1.4%, *p* = 0.52; surgical: 0.4% vs 0.4%, *p* = 0.60; and neoplasm: 6.0% vs 5.5%, *p* = 0.39).

The simple pre-post intervention analysis showed that the implementation of the shadowing programme had no significant impact on seven-day in-hospital mortality (Table 1). After adjustment for age, gender, comorbidities, small-area socioeconomic deprivation and year, there was no significant change in the odds ratio of death for the changeover vs control pre-intervention (OR = 1.03, 95% CI 0.97 to 1.09, *p* = 0.33) compared with post-intervention (OR = 1.06, 95% CI 0.98 to 1.14, *p* = 0.11). During the same period, there was no significant change for each clinical diagnosis category.

**Table 1:** Unadjusted/adjusted odds ratios and 95% confidence intervals before and after the implementation of the shadowing programme

Models	2003 to 2011		2012 to 2019	
	Odds ratio (95% CI)	***P*** value	Odds ratio (95% CI)	***P*** value
***All diagnosis***				
No covariates included	1.03 (0.97 to 1.10)	0.30	1.05 (0.98 to 1.12)	0.20
All covariates included	1.03 (0.97 to 1.09)	0.33	1.06 (0.98 to 1.14)	0.11
***Reallocated by primary diagnosis***				
***Medical***				
No covariates included	1.02 (0.96 to 1.10)	0.47	1.05 (0.97 to 1.14)	0.22
All covariates included	1.02 (0.95 to 1.09)	0.59	1.06 (0.97 to 1.15)	0.18
**Surgical**				
No covariates included	1.07 (0.91 to 1.25)	0.41	0.99 (0.83 to 1.18)	0.93
All covariates included	1.06 (0.90 to 1.25)	0.47	1.02 (0.86 to 1.21)	0.84
**Neoplasm**				
No covariates included	1.07 (0.90 to 1.26)	0.44	1.11 (0.93 to 1.32)	0.24
All covariates included	1.08 (0.91 to 1.27)	0.40	1.12 (0.94 to 1.33)	0.20

Table 2 and Table S2 (see Additional file 1) present the results of the generalised estimating equation model. For patients admitted on the first Wednesday in August, there was no significantly higher odds of death within 7 days after admission compared with patients admitted on the last Wednesday in July (OR = 1.03, 95% CI 0.94–1.13, *p* = 0.53). Furthermore, there was no significant change observed for any clinical diagnosis category.

**Table 2:** Results of generalised estimating equation analysis (odds ratio and its 95% confidence intervals) of seven-day in-hospital mortality between patients admitted to hospital on the first Wednesday in August compared with patients admitted on the last Wednesday in July

Outcome	OR (95% CI)	***p***-value
***Overall***		
Time	0.93 (0.92–0.94)	< 0.0001
Group (changeover group vs control group)	1.03 (0.97–1.09)	0.33
Intervention (after intervention vs before intervention)	1.04 (0.95–1.15)	0.39
Group*Intervention	1.03 (0.94–1.13)	0.53
***Reallocated by primary diagnosis***		
**Medical**		
Time	0.93 (0.92–0.95)	< 0.0001
Group (changeover group vs control group)	1.02 (0.95–1.09)	0.60
Intervention (after intervention vs before intervention)	1.01 (0.90–1.14)	0.84
Group*Intervention	1.04 (0.93–1.17)	0.48
**Surgical**		
Time	0.94 (0.91–0.96)	< 0.0001
Group (changeover group vs control group)	1.06 (0.90–1.25)	0.47
Intervention (after intervention vs before intervention)	0.99 (0.73–1.33)	0.93
Group*Intervention	0.96 (0.78–1.20)	0.96
**Neoplasm**		
Time	0.93 (0.90–0.96)	< 0.0001
Group (changeover group vs control group)	1.08 (0.91–1.28)	0.38
Intervention (after intervention vs before intervention)	1.45 (1.06–1.96)	0.02
Group*Intervention	1.04 (0.82–1.33)	0.75

## Discussion

In this observational study, we aimed to evaluate the association between the introduction of a national shadowing programme and in-hospital mortality during the first Wednesday in August. During the study period, the number of emergency admissions was increasing over time, though the mortality rates were declining. We found no significantly higher odds of death in mortality for patients admitted on the first Wednesday in August compared with patients admitted on the last Wednesday in July. Furthermore, we did not observe a significant change in either the medical, surgical or neoplasm diagnosis groups.

### Comparison with other studies

This is the first study that has assessed the impact of a shadowing programme on patient outcomes in healthcare though the evaluation of satisfaction of trainees was performed [16]. The programme is designed for final year medical students to observe an existing doctor undertaking the usual activities required of their role for 4 days before starting work. Only a few previous studies investigated the impact of the various induction programmes for junior doctors; however, all these studies reported only perceived benefits rather than changes in patient outcomes [17].

A significant amount of research investigated the association between the beginning of newly qualified doctors training and adverse effects on patient care [3]. The majority of these studies focused on 30-day or overall mortality for various subgroups of the patient [3, 18–20] or patients admitted to the intensive care unit [3, 21]. Only around 20% of these studies found an increase in mortality during the changeover, with an increase in relative risk between 4.3 and 41% or an adjusted odds ratio of 1.08 to 1.34.

Only two studies in the UK investigated the relationship between changeover and in-hospital mortality. Shuhaiber and colleagues [5] investigated the effect of cardiothoracic resident turnover on cardiac surgical outcomes and found a 30% higher odds of in-hospital mortality after a complex cardiac operation, but not for CABG alone. Jen et al. [6] found a 6% (OR = 1.06, 95% CI 1.00 to 1.13) higher mortality for patients admitted on the first Wednesday in August compared with patients on the last Wednesday in July [6]. Furthermore, they found a significant increase in mortality for medical patients (OR = 1.08, 95% CI 1.01 to 1.16, *p* = 0.03) but not for surgical or cancer patients. In comparison with Jen et al’s study, we found similar results for the pre-intervention period for all patients (1.03 vs. 1.06). However, in this study we looked at different time periods (Jen looked at 2000–8, and we looked at 2003–11). Interestingly, in our study, we found no significant increase in mortality for medical patients. In both studies, CCS diagnostic groups were used to divide admissions into three clinical categories; however, the proportions of medical and surgical diagnostic groups in our study (61.6 and 36.0%) were different from the one in Jen et al. work (85.1 and 12.1%). The reason why these proportions are different is not clear, but we were unable to replicate the groups exactly, which may explain different results.

### Strengths and limitations of the study

The main strength of our study is the use of a large and rich national administrative dataset that contains all hospital admissions and information relating to patient characteristics and some key outcomes such as in-hospital death. However, given that we focused on only 2 days of admissions a year and included only 7-day outcomes (to eliminate any potential overlap of care), we lacked power to detect small differences.

Another strength is the use of the generalised estimating equation method. The advantage of GEE models is that it provides unbiased estimation of population-level estimates despite the possible misspecification of the correlation structure. Furthermore, the use of GEE models with a control group enabled us to examine the effect of the shadowing programme despite the overall reduction in mortality in England (Fig. 2). The latter could make simple pre-post analysis potentially misleading.

Limitations mainly concern data availability. It is likely that a number of factors are responsible for the fall in in-hospital mortality over time, from changes in team mix and ways of working to discharge policies. For instance, there has been much work on failure to rescue following surgical complications [22], and teamwork is known to be important. Patient mix can change over time and can affect intervention effects if there is unmeasured confounding. Although the national hospital dataset includes information related to patient characteristics and the episode of care, ICD-10 is poor at capturing the severity of the disease. This could differ between the two patient groups, but its impact is likely to be modest. Furthermore, the dataset does not include any information related to intra-hospital transfers, which have been linked to various adverse outcomes, [22] or interpersonal and organisational dynamics within hospitals [23]. The programme may also have had an impact on other patient outcomes and have had educational benefits for staff that we could not assess.

Another potential factor that we were not able to account for was the emotional preparation of junior doctors. It has been shown that the start of a job in a new hospital can be a stressful time for junior doctors, which may affect the number of errors they make [24]. Moreover, every new medical member of staff has to attend organisational induction and mandatory training, which requires many doctors to be absent from patient care for a significant period [25].

## Conclusions

During the study period, we found no significant association between the introduction of shadowing programme and in-hospital mortality after adjusting for the pre-existing downward trend in mortality and available casemix factors; however, methodological limitations limit the power of the study and we cannot rule out an effect of the shadowing programme on other outcomes.

## Supplementary Information

**Additional file 1.**

## Data Availability

The data that support the findings of this study are available from NHS DIGITAL in England but restrictions apply to the availability of these data, which were used under agreement between NHS DIGITAL and Dr. Foster Unit at Imperial, and so are not publicly available. The authors are not in a position to release the data (in accordance with the agreement with NHS DIGITAL). The data can be access via NHS Digital, all information who can access data and how to apply for the access can be found here: https://digital.nhs.uk/data-and-information/data-tools-and-services/data-services/hospital-episode-statistics/users-uses-and-access-to-hospital-episode-statistics
