# Supplementary material for: Assessing the impact of a shadowing programme on in-hospital mortality following trainee doctors’ changeover
Source: BMC Health Serv Res. 2021 Jun 7;21:563. doi: 10.1186/s12913-021-06578-y (PMC8186218; doi:10.1186/s12913-021-06578-y)
Supplement: Supplementary file 1 — Additional file 1. [file 12913_2021_6578_MOESM1_ESM.docx]

Assessing the impact of a shadowing programme on in-hospital mortality following trainee doctors’ changeover

Balinskaite Violeta, Bottle Alex and Aylin Paul

Table S1 Characteristics of the study population

|  | Emergency admissions on the last Wednesday in July | | | | Emergency admissions on the first Wednesday in August | | | |
| --- | --- | --- | --- | --- | --- | --- | --- | --- |
|  | 2003 to 2011 | | 2012 to 2019 | | 2003 to 2011 | | 2012 to 2019 | |
|  | Admissions  (% of total) | Deaths  (in-hospital) | Admissions  (% of total) | Deaths  (in-hospital) | Admissions  (% of total) | Deaths  (in-hospital) | Admissions  (% of total) | Deaths  (in-hospital) |
| Total | 159435 | 2058 | 165872 | 1714 | 155759 | 2075 | 162952 | 1765 |
| Year |  |  |  |  |  |  |  |  |
| 2003 | 14932  (9.4) | 226 |  |  | 14826  (9.5) | 269 |  |  |
| 2004 | 15910  (10.0) | 239 |  |  | 15615  (10.0) | 252 |  |  |
| 2005 | 16492  (10.3) | 222 |  |  | 16442  (10.6) | 226 |  |  |
| 2006 | 17531  (11.0) | 246 |  |  | 16779  (10.8) | 217 |  |  |
| 2007 | 18139  (11.4) | 235 |  |  | 17152  (11.0) | 247 |  |  |
| 2008 | 18532  (11.6) | 234 |  |  | 17917  (11.5) | 225 |  |  |
| 2009 | 18618  (11.7) | 215 |  |  | 18893  (12.1) | 215 |  |  |
| 2010 | 19531  (12.3) | 221 |  |  | 18835  (12.1) | 208 |  |  |
| 2011 | 19750  (12.4) | 220 |  |  | 19300  (12.4) | 216 |  |  |
| 2012 |  |  | 20388  (12.3) | 234 |  |  | 19965  (12.3) | 227 |
| 2013 |  |  | 19630  (11.8) | 198 |  |  | 19274  (11.8) | 237 |
| 2014 |  |  | 20212  (12.2) | 197 |  |  | 19773  (12.1) | 195 |
| 2015 |  |  | 19960  (12.0) | 203 |  |  | 19727  (12.1) | 239 |
| 2016 |  |  | 20886  (12.6) | 212 |  |  | 20390  (12.5) | 207 |
| 2017 |  |  | 21194  (12.8) | 215 |  |  | 20787  (12.8) | 239 |
| 2018 |  |  | 21470  (12.9) | 231 |  |  | 21204  (13.0) | 208 |
| 2019 |  |  | 22132  (13.3) | 224 |  |  | 21830  (13.4) | 213 |
| Age |  |  |  |  |  |  |  |  |
| 0-14 | 29775  (18.7) | 127  (6.2) | 29552  (17.8) | 83  (4.8) | 28603  (18.4) | 101  (4.9) | 27953  (17.2) | 74  (4.2) |
| 15-44 | 62473  (39.2) | 47  (2.3) | 57168  (34.5) | 44  (2.6) | 61035  (39.2) | 61  (2.9) | 56687  (34.8) | 47  (2.7) |
| 45-64 | 23021  (14.4) | 249  (12.1) | 26353  (15.9) | 218  (12.7) | 22652  (14.5) | 244  (11.8) | 26267  (16.1) | 241  (13.7) |
| 65-74 | 14647  (9.2) | 357  (17.4) | 17107  (10.3) | 325  (19.0) | 14217  (9.1) | 361  (17.4) | 17144  (10.5) | 309  (17.5) |
| 75-84 | 18014  (11.3) | 656  (31.9) | 20279  (12.2) | 508  (29.6) | 17736  (11.4) | 711  (34.3) | 19778  (12.1) | 520  (29.5) |
| 85+ | 11505  (7.2) | 622  (30.2) | 15413  (9.3) | 536  (31.3) | 11516  (7.4) | 597  (28.8) | 15123  (9.3) | 574  (32.5) |
| Gender |  |  |  |  |  |  |  |  |
| Male | 61982  (38.9) | 1019  (49.5) | 67134  (40.5) | 896  (52.3) | 60425  (38.8) | 1020  (49.2) | 65368  (40.1) | 897  (50.8) |
| Female | 97453  (61.1) | 1039  (50.5) | 98738  (59.5) | 818  (47.7) | 95334  (61.2) | 1055  (50.8) | 97584  (59.9) | 868  (49.2) |
| Index of multiple deprivation quintile |  |  |  |  |  |  |  |  |
| 1 (least deprived) | 21272  (13.3) | 273  (13.3) | 24256  (14.6) | 273  (15.9) | 20688  (13.3) | 291  (14.0) | 23978  (14.7) | 311  (17.6) |
| 2 | 24249  (15.2) | 359  (17.4) | 27183  (16.4) | 330  (19.3) | 23731  (15.2) | 405  (19.5) | 26861  (16.5) | 362  (20.5) |
| 3 | 27536  (17.3) | 417  (20.3) | 29321  (17.7) | 359  (21.0) | 26792  (17.2) | 435  (21.0) | 28897  (17.7) | 356  (20.2) |
| 4 | 31292  (19.6) | 458  (22.3) | 31989  (19.3) | 347  (20.3) | 30680  (19.7) | 425  (20.5) | 31263  (19.2) | 344  (19.5) |
| 5 (most deprived) | 37778  (23.7) | 412  (20.0) | 36723  (22.1) | 317  (18.5) | 36998  (23.8) | 407  (19.6) | 36056  (22.1) | 309  (17.5) |
| 6 (unknown) | 17308  (10.9) | 139  (6.8) | 16400  (9.9) | 88  (5.1) | 16870  (10.8) | 112  (5.4) | 15897  (9.8) | 83  (4.7) |
| Charlson index of comorbidity (HES adaptation) |  |  |  |  |  |  |  |  |
| 0 | 123249  (77.3) | 718  (34.9) | 110497  (66.6) | 376  (21.9) | 120499  (77.4) | 781  (37.6) | 108488  (66.6) | 413  (23.4) |
| 0-4 | 13519  (8.5) | 204  (9.9) | 18447  (11.1) | 178  (10.4) | 13187  (8.5) | 196  (9.5) | 18611  (11.4) | 179  (10.1) |
| 5-9 | 7649  (4.8) | 237  (11.5) | 9605  (5.8) | 144  (8.4) | 7310  (4.7) | 199  (9.6) | 9402  (5.8) | 162  (9.2) |
| 10-14 | 8236  (5.2) | 455  (22.1) | 12282  (7.4) | 366  (21.4) | 8039  (5.2) | 449  (21.6) | 12047  (7.4) | 352  (19.9) |
| 15-24 | 4968  (3.1) | 291  (14.1) | 9837  (5.9) | 392  (22.9) | 5022  (3.2) | 315  (15.2) | 9460  (5.8) | 377  (21.4) |
| 25+ | 1814  (1.1) | 153  (7.4) | 5204  (3.1) | 258  (15.1) | 1702  (1.1) | 135  (6.5) | 4944  (3.0) | 282  (16.0) |
| Diagnosis groups |  |  |  |  |  |  |  |  |
| Medical | 96289  (60.4) | 1546  (75.1) | 104581  (63.0) | 1318  (76.9) | 93902  (60.3) | 1545  (74.5) | 102374  (62.8) | 1358  (76.9) |
| Surgical | 58961  (37.0) | 270  (13.1) | 57776  (34.8) | 214  (12.5) | 57842  (37.1) | 283  (13.6) | 57148  (35.1) | 211  (12.0) |
| Neoplasm | 4185  (2.6) | 242  (11.8) | 3515  (2.1) | 182  (10.6) | 4015  (2.6) | 247  (11.9) | 3430  (2.1) | 196  (11.1) |

Table S2. Results of generalised estimating equation analysis (odds ratio and its 95% confidence intervals) of seven-day in-hospital mortality between patients admitted to hospital on the first Wednesday in August compared with patients admitted on the last Wednesday in July

| Outcome | OR (95% CI) | p-value |
| --- | --- | --- |
| *Overall* |  |  |
| *Gender* |  |  |
| Male | 1.15 (1.10-1.20) | <0.0001 |
| Female | 1 |  |
| *Age* |  | 0.28 |
| 0-14 | 2.18 (1.82-2.61) |  |
| 15-44 | 1 |  |
| 45-64 | 8.70 (7.36 – 10.28) |  |
| 65-74 | 15.73 (13.16-18.79) |  |
| 75-84 | 21.15 (17.76-25.19) |  |
| 85+ | 28.54 (24.01-33.93) |  |
| Index of multiple deprivation quintile |  | <0.0001 |
| 1 (least deprived) | 1 |  |
| 2 | 1.06 (0.98 – 1.15) |  |
| 3 | 1.08 (1.00-1.17) |  |
| 4 | 1.08 (1.00-1.17) |  |
| 5 (most deprived) | 1.01 (0.93 – 1.09) |  |
| 6 (unknown) | 2.79 (2.42-3.22) |  |
| Charlson index of comorbidity (HES adaptation) |  | <0.0001 |
| 0 | 1 |  |
| 0-4 | 1.37 (1.25-1.49) |  |
| 5-9 | 1.82 (1.65-2.01) |  |
| 10-14 | 2.93 (2.69-3.19) |  |
| 15-24 | 3.60 (3.32-3.89) |  |
| 25+ | 4.63 (4.20-5.12) |  |
| *Reallocated by primary diagnosis* |  |  |
| *Medical* |  |  |
| Gender |  | <0.0001 |
| Male | 1.07 (1.02-1.12) |  |
| Female | 1 |  |
| Age |  | <0.0001 |
| 0-14 | 0.70 (0.55-0.89) |  |
| 15-44 | 1 |  |
| 45-64 | 4.85 (4.01-5.87) |  |
| 65-74 | 8.89 (7.28-10.85) |  |
| 75-84 | 12.80 (10.48-15.63) |  |
| 85+ | 18.49 (15.11-22.62) |  |
| Index of multiple deprivation quintile |  | 0.70 |
| 1 (least deprived) | 1 |  |
| 2 | 0.99 (0.91-1.09) |  |
| 3 | 1.03 (0.94-1.12) |  |
| 4 | 1.03 (0.94-1.13) |  |
| 5 (most deprived) | 0.99 (0.90-1.08) |  |
| 6 (unknown) | 2.05 (1.69-2.48) |  |
| Charlson index of comorbidity (HES adaptation) |  | <0.0001 |
| 0 | 1 |  |
| 0-4 | 1.33 (1.21-1.47) |  |
| 5-9 | 1.73 (1.55-1.93) |  |
| 10-14 | 2.58 (2.35-2.84) |  |
| 15-24 | 3.19 (2.91-3.51) |  |
| 25+ | 4.17 (3.75-4.64) |  |
| *Surgical* |  |  |
| Gender |  | 0.0016 |
| Male | 1.26 (1.09-1.46) |  |
| Female | 1 |  |
| Age |  | <0.0001 |
| 0-14 | 14.50 (9.52-22.06) |  |
| 15-44 | 1 |  |
| 45-64 | 19.95 (6.43-15.40) |  |
| 65-74 | 23.09 (15.00-35.53) |  |
| 75-84 | 44.99 (29.62-68.35) |  |
| 85+ | 62.03 (41.45-92.83) |  |
| Index of multiple deprivation quintile |  | 0.215 |
| 1 (least deprived) | 1 |  |
| 2 | 1.35(1.05-1.75) |  |
| 3 | 1.40 (1.07-1.83) |  |
| 4 | 1.39 (1.09-1.77) |  |
| 5 (most deprived) | 0.97 (0.73-1.29) |  |
| 6 (unknown) | 8.41 (6.28-11.27) |  |
| Charlson index of comorbidity (HES adaptation) |  | <0.0001 |
| 0 | 1 |  |
| 0-4 | 1.39 (1.10-1.77) |  |
| 5-9 | 2.21 (1.67-2.92) |  |
| 10-14 | 2.43 (1.95-3.03) |  |
| 15-24 | 3.69 (2.93-4.65) |  |
| 25+ | 4.24 (3.15-5.71) |  |
| *Neoplasm* |  |  |
| Gender |  | 0.0008 |
| Male | 1.28 (1.11-1.48) |  |
| Female | 1 |  |
| Age |  | <0.0001 |
| 0-14 | 0.23 (0.05-0.14) |  |
| 15-44 | 1 |  |
| 45-64 | 2.58 (2.62-4.11) |  |
| 65-74 | 3.46 (2.18-5.48) |  |
| 75-84 | 3.62 (2.31-5.67) |  |
| 85+ | 3.51 (2.16-5.70) |  |
| Index of multiple deprivation quintile |  | 0.328 |
| 1 (least deprived) | 1 |  |
| 2 | 1.22 (0.95-1.56) |  |
| 3 | 1.15 (0.89-1.48) |  |
| 4 | 1.14 (0.85-1.51) |  |
| 5 (most deprived) | 1.04 (0.81-1.35) |  |
| 6 (unknown) | 0.41 (0.14-1.24) |  |
| Charlson index of comorbidity (HES adaptation) |  | 0.012 |
| 0 | 1 |  |
| 0-4 | 1.48 (1.08-2.03) |  |
| 5-9 | 1.51 (1.10-2.06) |  |
| 10-14 | 2.73 (2.27-3.28) |  |
| 15-24 | 3.28 (2.72-3.95) |  |
| 25+ | 4.71 (3.62-6.14) |  |
